# Weakly supervised deep learning for determining the prognostic value of ^18^F-FDG PET/CT in extranodal natural killer/T cell lymphoma, nasal type

**DOI:** 10.1007/s00259-021-05232-3

**Published:** 2021-02-20

**Authors:** Rui Guo, Xiaobin Hu, Haoming Song, Pengpeng Xu, Haoping Xu, Axel Rominger, Xiaozhu Lin, Bjoern Menze, Biao Li, Kuangyu Shi

**Affiliations:** 1 Department of Nuclear Medicine, Ruijin Hospital, Shanghai Jiao Tong University School of Medicine, Shanghai, China; 2 Department of Informatics, Technical University of Munich, Munich, Germany; 3 State Key Laboratory of Medical Genomics, Shanghai Institute of Hematology, Ruijin Hospital, Shanghai Jiao Tong University School of Medicine, Shanghai, China; 4 Department of Radiation, Ruijin Hospital, Shanghai Jiao Tong University School of Medicine, Shanghai, China; 5 Department of Nuclear Medicine, University of Bern, Bern, Switzerland; 6 Department of Quantitative Biomedicine, University of Zurich, Zurich, Switzerland

**Keywords:** Deep learning, ^18^F-FDG PET/CT, Extranodal natural killer/T cell lymphoma, Prognosis, Progression-free survival

## Abstract

**Purpose:**

To develop a weakly supervised deep learning (WSDL) method that could utilize incomplete/missing survival data to predict the prognosis of extranodal natural killer/T cell lymphoma, nasal type (ENKTL) based on pretreatment ^18^F-FDG PET/CT results.

**Methods:**

One hundred and sixty-seven patients with ENKTL who underwent pretreatment ^18^F-FDG PET/CT were retrospectively collected. Eighty-four patients were followed up for at least 2 years (training set = 64, test set = 20). A WSDL method was developed to enable the integration of the remaining 83 patients with incomplete/missing follow-up information in the training set. To test generalization, these data were derived from three types of scanners. Prediction similarity index (PSI) was derived from deep learning features of images. Its discriminative ability was calculated and compared with that of a conventional deep learning (CDL) method. Univariate and multivariate analyses helped explore the significance of PSI and clinical features.

**Results:**

PSI achieved area under the curve scores of 0.9858 and 0.9946 (training set) and 0.8750 and 0.7344 (test set) in the prediction of progression-free survival (PFS) with the WSDL and CDL methods, respectively. PSI threshold of 1.0 could significantly differentiate the prognosis. In the test set, WSDL and CDL achieved prediction sensitivity, specificity, and accuracy of 87.50% and 62.50%, 83.33% and 83.33%, and 85.00% and 75.00%, respectively. Multivariate analysis confirmed PSI to be an independent significant predictor of PFS in both the methods.

**Conclusion:**

The WSDL-based framework was more effective for extracting ^18^F-FDG PET/CT features and predicting the prognosis of ENKTL than the CDL method.

**Supplementary Information:**

The online version contains supplementary material available at 10.1007/s00259-021-05232-3.

## Introduction

The emergence of artificial intelligence (AI) in the field of medical imaging has led to several breakthroughs [1, 2]. AI has already proven to be advantageous for computer-aided diagnosis in medical imaging, such as for the differential diagnosis of coronavirus disease 2019 [3], skin cancer [4], and diabetic retinopathy [5]. Moreover, it has been developed to help identify imaging-based biomarkers, leading to an improvement in the prognosis of, for example, lung cancer [6, 7], gliomas [8], and nasopharynx cancer [9]. Deep learning is an indispensable part of AI and has been reported to be extremely effective in several medical imaging-related tasks, such as image segmentation, registration, fusion, annotation, computer-aided diagnosis and prognosis analyses, lesion and landmark detection, and microscopic imaging analysis. In such studies, deep learning networks have shown capabilities to automatically extract characteristic features from images, including explicit features, such as the location, distribution, and volume size of lesions, and implicit features at different levels, which were deduced using nonlinear, independent discriminant, and invariant properties. The end-to-end automatic feature extraction does not involve human interaction, and the extracted features are the most implicit. Although the implicit features may be difficult to interpret, they are determinant for the performance of convolutional neural networks (CNNs) and play critical roles in many medical applications [10, 11].

The development of deep learning depends on the availability of a huge amount of data. It is usually challenging to gather a large cohort of patients with survival follow-up after administering the same therapeutic regime. Clinical trials are often associated with incomplete or missing follow-up due to factors such as insufficient follow-up time, patient tolerance, and compliance. This consequently hampers extensive development of deep learning methods for predicting therapeutic prognosis. Maximizing the utility of data gathered by clinical trials is thus a key area of research.

Data augmentation methods such as deformation or generative adversarial networks are often applied to support the development of deep learning methods in the field of image analysis [12]. However, the relationship among imaging, therapy, and survival is more complex than general image analyses. The increased physiological complexity makes it difficult to synthesize meaningful data for training. Furthermore, errors in data preparation may mislead algorithmic development [13]. Weakly supervised classification methods have been established using unlabeled data for regularization under particular distributional assumptions, such as cluster or smoothness assumption; however, the performance relies on the fidelity of the assumption [14–16], and it is usually challenging to find a proper assumption in real application. In contrast, positive–negative unlabeled (PNU) classification [15] is a weakly supervised strategy to deal with a tough task with less knowledge regarding data distribution and, therefore, is less restricted in complex applications. Despite these advantages, because PNU classification is generally applied for classification problems based on low-dimensional feature vectors [15], it is not straightforward to apply this classification to imaging data for survival follow-up in order to improve therapeutic prognosis.

Extranodal natural killer/T cell lymphoma, nasal type (ENKTL) is a rare type of lymphoma with poor survival outcome [17–19]. It constitutes <1% of all lymphomas in Western countries and 3–9% of all malignant lymphomas in Asia [18, 20, 21]. Several investigations have identified that almost all ENKTL lesions are fluorodeoxyglucose (FDG) avid [22, 23]. In patients with ENKTL, the use of ^18^F-FDG positron emission tomography/computed tomography (PET/CT) for staging is widespread [24–26]. Nevertheless, many contradictions exist pertaining to the value of ^18^F-FDG PET/CT in predicting the prognosis of ENKTL [22, 27–30]. Some studies [31, 32] have reported that maximum standardized uptake value (SUVmax) of pretreatment ^18^F-FDG PET/CT is not a statistically significant predictor of overall survival and progression-free survival (PFS). Tumor ^18^F-FDG uptake cannot reflect the aggressive biologic behavior of ENKTL; however, some studies have reported contradictory results [30, 33]. These studies found that high tumor ^18^F-FDG uptake was closely associated with unfavorable treatment and survival outcomes. Chang et al. [34] reported that baseline whole-body total lesion glycolysis (TLG) was a good predictor of PFS and overall survival in patients with ENKTL. However, treatment plans were not uniform in these studies, potentially affecting the treatment outcome and predictive value of pretreatment ^18^F-FDG PET/CT. Prospective research methods have also been used to assess the prognostic value of ^18^F-FDG PET/CT in ENKTL [31, 35, 36], but considering some uncertainty in the reported results, it remains unclear. A novel solution is accordingly needed. Although deep learning has been advantageous in assisting molecular imaging to optimize therapeutic prognosis [9], it is extremely difficult to develop appropriate deep learning methods for this rare condition with only a limited number of cases.

We herein propose a weakly supervised deep learning (WSDL) method based on PNU classification to maximize the utility of incomplete and missing follow-up data so as to predict the prognosis of ENKTL. We investigated the accuracy and robustness of this data enhancement strategy on a retrospective cohort to test a therapeutic regime for ENKTL.

## Material and methods

### Patients

One hundred and sixteen-seven patients with histopathologically diagnosed ENKTL from June 2011 to October 2020 recruited at Shanghai Ruijin Hospital were retrospectively collected. Patients who had undergone surgical resection, radiotherapy, chemotherapy, and/or bone marrow transplantation as well as those with other malignancies were excluded. All patients underwent whole-body ^18^F-FDG PET/CT for initial staging before therapy and were then treated with a therapeutic regime of methotrexate, etoposide, dexamethasone, and pegaspargase (MESA). Eighty-four patients were followed up for at least 2 years. Among them, 49 were sandwiched with radiotherapy for the involved local focus 21 days after two cycles of MESA. They were treated with a linear accelerator producing 6 MV photons. The radiotherapy dose was 50 Gy in 25 fractions, once a day, and 5 fractions every week. Chemotherapy was restarted 28 days after radiotherapy.

**Fig. 1:**
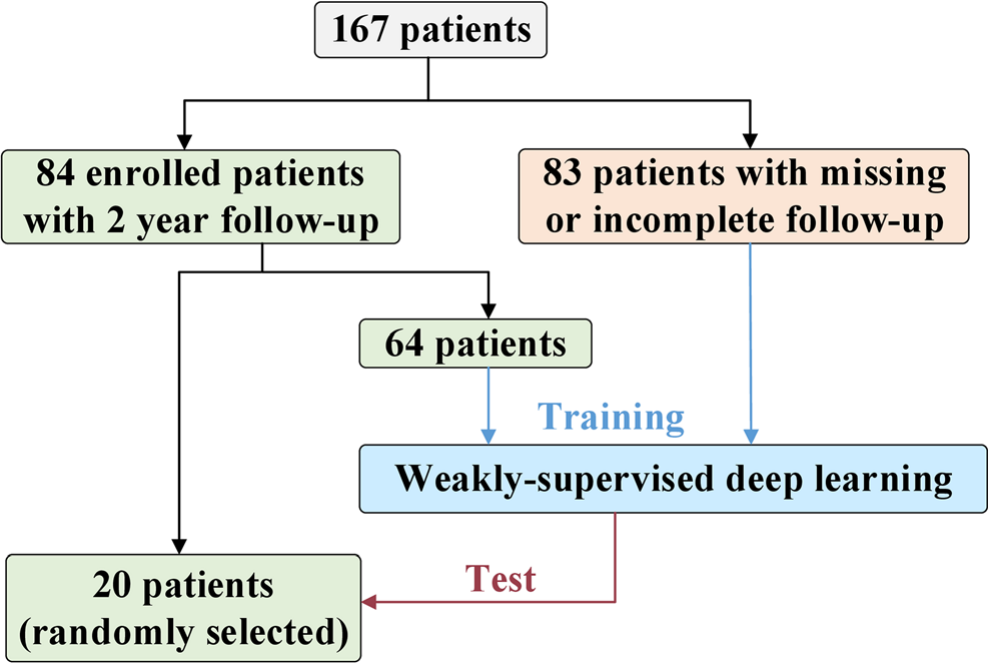
A flow chart depicting the study plan. ENKTL: extranodal natural killer/T cell lymphoma, nasal type

Of the 84 patients, 64 were randomly included in the training set; the remaining 20 were unobserved and included in the test set. The ratio of relapse to non-relapse individuals was kept the same in the test and training sets to avoid an extreme imbalance problem. PFS was the major endpoint. Recurrence and lymphoma infiltration were mainly diagnosed based on imaging methods and pathology. The remaining 83 patients without follow-up information or followed up for <2 years were also included in the training set using the proposed WSDL method. To further test the generalization of the WSDL method, data pertaining to the 83 patients were derived from three types of scanners: Scanner 1 (Discovery VCT, GE Healthcare, USA, 39 patients), 2 (Discovery MI, GE Healthcare, USA, 29 patients), and 3 (Biograph Vision, SIEMENS, Germany, 15 patients). The training set thus ultimately comprised 147 patients (Fig. 1).

The clinical features of the 84 patients, including gender, age, serum lactate dehydrogenase levels, Eastern Cooperative Oncology Group (ECOG) score, Ki67, β2-microglobulin, Epstein–Barr virus DNA, and B symptoms, were recorded. Ann Arbor stage, SUVmax, mean SUV (SUVmean), metabolic tumor volume (MTV), and TLG extracted from ^18^F-FDG PET/CT were also measured. All procedures in the study were performed in accordance with the ethical standards of the committee from Ruijin Hospital, Shanghai Jiao Tong University, School of Medicine. Written informed consent was obtained from all patients before treatment. Among the 84 patients enrolled in the clinical trial, 58 were alive (12 presented with persistent or recurrent disease at the last follow-up), and 26 had died due to a tumor-related disease. The clinical characteristics of patients in the training and test sets have been summarized in Table 1; data pertaining to the 83 patients diagnosed with ENKTL but with missing or incomplete follow-up information are also listed.

**Table 1:** Clinical characteristics of patients

Characteristics	Training cohort (*n* = 64), no. (%)	Test cohort (*n* = 20), no. (%)	*P*	Patients with missing or incomplete data (*n* = 83), no. (%)		
				Scanner 1 (*n* = 39)	Scanner 2 (*n* = 29)	Scanner 3 (*n* = 15)
*Gender			0.690			
Male	45 (70.31)	15 (75.00)		28 (71.79)	21 (72.41)	10 (66.67)
Female	19 (29.69)	5 (25.00)		11 (28.21)	8 (27.59)	5 (33.33)
*Age (years)			0.861			
< 60	50 (78.13)	16 (80.00)		24 (61.54)	22 (75.86)	10 (66.67)
≥ 60	14 (21.87)	4 (20.00)		15 (38.46)	7 (24.14)	5 (33.33)
*Primary site of tumor			0.078			
Upper aerodigestive tract	51 (79.69)	12 (60.00)		30 (76.92)	25 (86.21)	13 (86.67)
Non-upper aerodigestive tract	13(20.31)	8 (40.00)		9 (23.08)	4 (13.79)	2 (13.33)
*Ann Arbor stage			0.182			
I–II	51 (79.69)	13 (65.00)		30 (76.92)	23 (79.31)	11 (73.33)
III–IV	13(20.31)	7 (35.00)		9 (23.08)	6 (20.69)	4 (26.67)
*B symptoms			0.213			
Yes	25 (39.06)	9 (45.00)		–	–	–
No	39 (60.94)	11 (55.00)		–	–	–
*ECOG score			0.038			
0	36 (56.25)	6 (30.00)		–	–	–
1	19 (29.69)	7 (35.00)		–	–	–
2–5	9 (14.06)	7 (35.00)		–	–	–
*PINK			0.230			
Low risk (0)	37 (57.81)	11 (55.00)		–	–	–
Intermediate risk (1)	16 (25.00)	1 (5.00)		–	–	–
High risk (2–4)	11 (17.19)	8 (40.00)		–	–	–
**^18^F-FDG uptake (SUVmax)	13.17 ± 6.90	14.48 ± 4.92	0.432	12.01 ± 6.07	16.40 ± 6.78	21.23 ± 9.06
**Follow-up period (months)	33.70 ± 20.82	38.70 ± 23.96	0.369	–	–	–

*P values were calculated using the chi-squared test for categorical variables and nonparametric test for continuous variables

**Mean ± SD; independent sample *t* test was used to compare differences in quantitative parameters between the groups

Abbreviations: LDH, lactate dehydrogenase; ECOG, Eastern Cooperative Oncology Group; PINK, prognostic index of natural killer lymphoma; SUVmax: maximum standardized uptake value

### ^18^F-FDG PET/CT and preprocessing

Patients were required to fast for at least 6 h before ^18^F-FDG PET/CT, and the serum glucose level was maintained under 7.0 mmol/L. Whole-body PET from the head to thigh was performed 1 h after intravenously administering 5–6 MBq of ^18^F-FDG per kilogram of body weight. In case of Scanner 1, PET was performed in the 3D mode with an acquisition time of 2 min per bed position covering the same field as the CT scan. CT was performed using the following parameters: 120–180 mA, 140 kV, gantry rotation speed of 0.8 s, and thick axial section of 3.75 mm. After correcting attenuation (based on CT), scatter, dead time, and random coincidences, PET images were reconstructed using 3D ordered-subset expectation maximization (OSEM) with a Gaussian filter (full width at half maximum of 6 mm), leading to images with voxel size of 5.47 mm. In case of Scanner 2, PET was performed in the 3D mode with an acquisition time of 1.5 min per bed position covering the same field as the CT scan. CT was performed using the following parameters: 120–180 mA, 140 kV, and gantry rotation speed of 0.8 s. PET images were reconstructed using the block-sequential regularized expectation maximization reconstruction algorithm (Q.clear, GE Healthcare, USA), which had a *β* value of 550 with a 256 × 256 matrix (pixel size = 2.7 × 2.7 mm^2^, slice thickness = 2.79 mm). Finally, in case of Scanner 3, CT was performed using the following parameters: 146 mA, 120 kV, and spiral pitch factor of 1. Images were reconstructed using the 3D ordinary Poisson OSEM algorithm, with four iterations and five subsets, application of time-of-flight resolution modeling, and no filtering. The obtained PET images had an image matrix of 440 × 440, pixel size of 1.6 × 1.6 × 1.5 mm, and slice thickness of 2.0 mm. Lymphoma lesions in the training set were manually delineated on the fusion map of PET/CT images using ITK-SNAP (v3.6.0) by a nuclear medicine physician with 15 years of experience [9].

### WSDL for feature extraction

The WSDL method based on Residual Network-18 (ResNet-18) [37] was proposed to predict disease prognosis using a well-exploiting unlabeled dataset (83 patients without follow-up information). The summarized algorithm for the WSDL method is as follows:

- **Input**: 3D volumetric image I of size width × height × depth
- **Ensure**: Image I is a rank 3 tensor

1. Train deep convolutional neural networks (DCNNs) with labeled data to obtain the baseline model
2. Use baseline DCNNs to extract features from labeled and unlabeled data
3. Build the PNU classifier to generate implicit labels for unlabeled data
4. Re-train DCNNs with labeled and unlabeled data to obtain the final prognosis

The ResNet is an artificial neural network that is inspired by the biological neural networks constituting animal brains. DCNNs were constructed for deep learning feature extraction. They are a simplified version of ResNet-18 and were implemented using the Python Keras package with TensorFlow as the backend. The 83 patients with missing or incomplete follow-up data were included in the training set along with 64 patients with follow-up data. Labels for the 83 patients were implicitly derived using the PNU classifier during the training procedure, leading to maximized prediction probability. Further details are provided in Supplementary Materials.

**Fig. 2:**
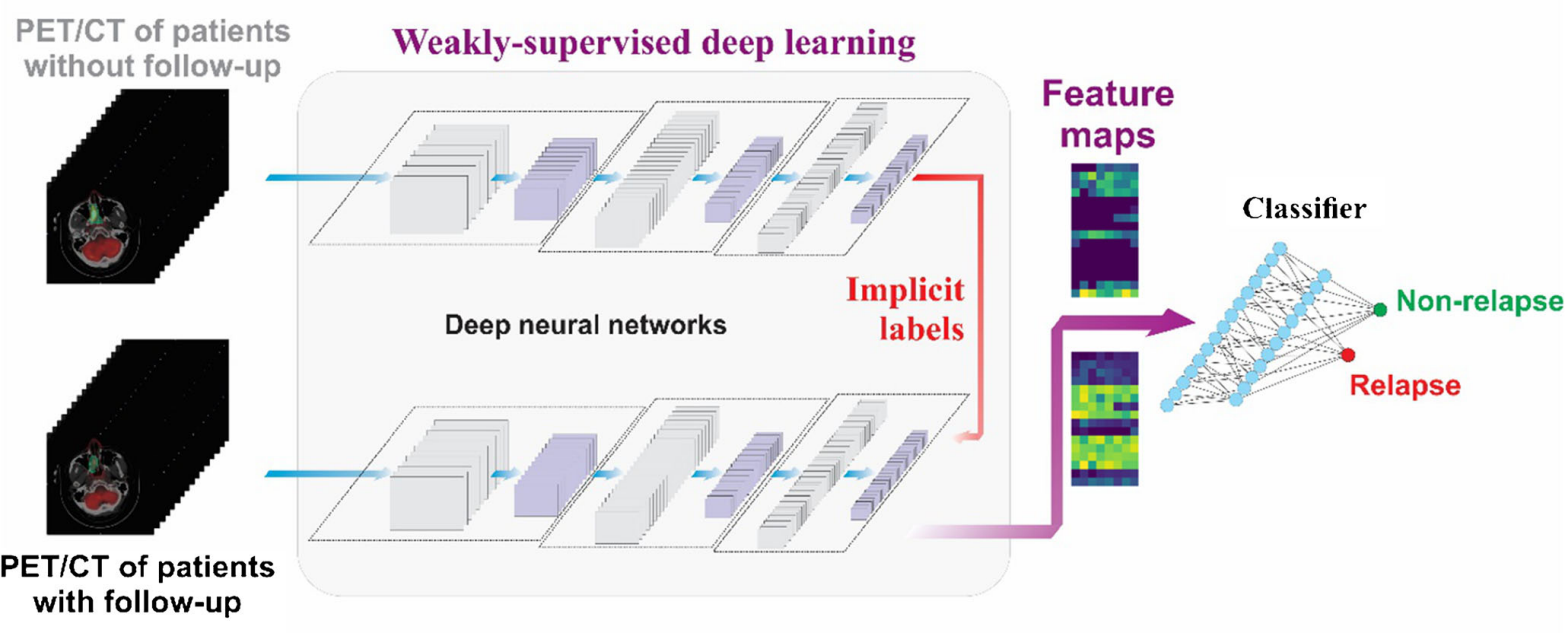
An illustration of the concept of the proposed weakly supervised deep learning method

In total, 128 deep learning features were extracted from the output of the average pooling layer of DCNNs for PET/CT images in the training set, which were grouped into a 16 × 8 feature map for visualization. We herein propose a new biomarker in the form of prediction similarity index (PSI), which is the ratio of the positive predicted probability value to the negative predicted probability value. It was derived from these features to predict the probability of recurrence and non-recurrence. PSI of 1 was used to differentiate between positive and negative predictions. To determine the advantages of the WSDL method, we compared it with the conventional deep learning (CDL) method of our proposed DCNNs trained only on the 64 patients followed up for at least 2 years (Fig. 2).

### Statistics

SPSS v23.0 (SPSS Inc., Chicago, IL, USA) and GraphPad Prism 8.0.1 (GraphPad, San Diego, USA) were used for statistical analyses. Univariate analysis using the Kaplan–Meier method was performed for each variable with a potential prognostic value. Time-dependent receiver operating characteristic (ROC) analysis was performed to evaluate the discriminative ability of PSI for the prognostic prediction of ENKTL. PSI-based PFS, prediction sensitivity and specificity, and accuracy of PSI were calculated. Differences in sensitivity and specificity between the WSDL and CDL methods were compared using the Fisher’s exact test. The log-rank test was used to compare differences in PFS between the groups (PSI > 1 and PSI < 1). Multivariate analysis using the Cox proportional hazards model was used to assess the independent effects of PSI and clinical parameters of the disease. *P* < 0.05 indicated statistical significance.

## Results

### Extraction of deep learning features

**Fig. 3:**
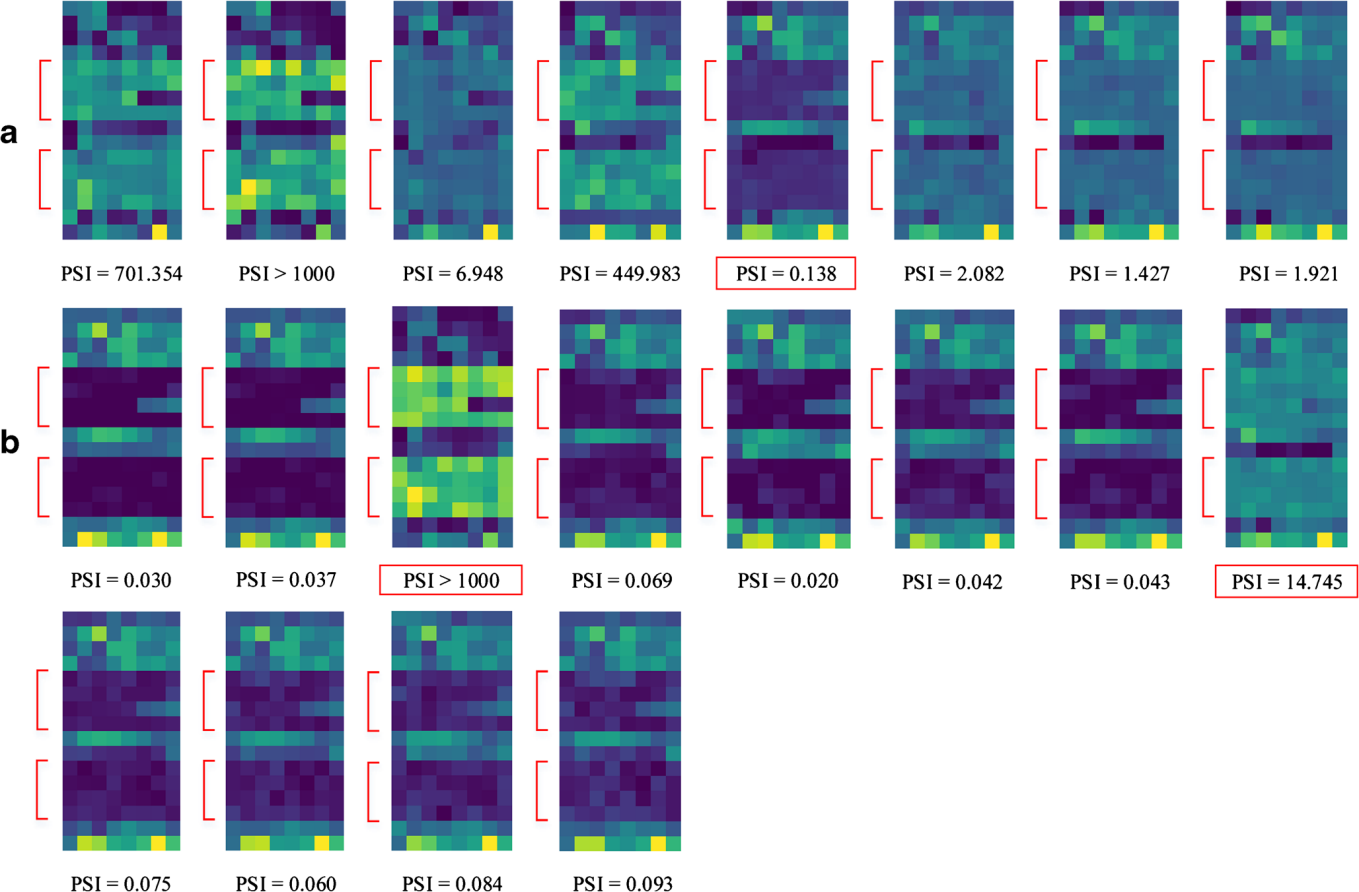
Visualization of the feature maps (16 × 8) representing 128 features extracted by the proposed WSDL method in the test set. Each strip represents the feature map of a patient. Red arrows indicate the characteristic difference between the (A) relapse and (B) non-relapse groups in the test cohort. PSI results with incorrect predictions have been marked by red boxes

One hundred and twenty-eight features were extracted from tumor ROIs outlined on ^18^F-FDG PET/CT scans of each patient using the proposed WSDL method. These ROIs were outlined based on lesion locations and shapes, while non-meaningful background was cut off. The 128 features were grouped into feature maps of 16 × 8 strips. The feature maps of the test set (*n* = 20) have been illustrated in Fig. 3. In general, characteristic differences between relapse and non-relapse patients could be visualized on these maps. The feature maps of the training set (*n* = 64) have been illustrated in Supplementary Figure S1 (relapse) and S2 (non-relapse), whereas those of the 83 patients with incomplete or missing follow-up data and who were imaged using the aforementioned scanners are illustrated in Figure S3. The feature maps of the test set (Figure S4) and training set (Figure S5 for relapse, Figure S6 for non-relapse) with the CDL method have also been illustrated in supplementary figures.

### PSI as the prognostic score

**Fig. 4:**
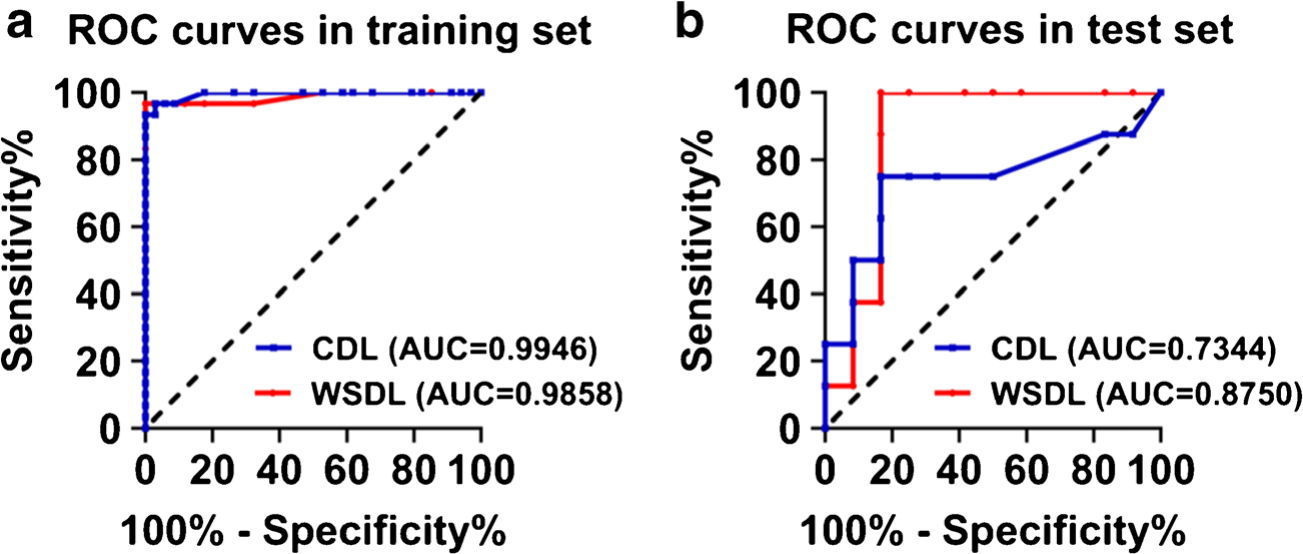
ROC curves comparing the predictive power of PSI for PFS in the training (A) and test (B) sets. ROC, receiver operator characteristic; AUC, area under the curve; PSI, prediction similarity index; WSDL, weakly supervised deep learning; CDL, conventional deep learning

Patients with PSI > 1 were considered to show a positive response, while those with PSI < 1 were considered to show a negative response. The ROC curves of the results of the WSDL and CDL methods were compared (Fig. 4). With the WSDL method, in the training and test sets, PSI achieved area under the curve (AUC) scores of 0.986 (*P* = 0.000, 95% CI, 0.957–1.000) and 0.875 (*P* = 0.005, 95% CI, 0.706–1.000), respectively, in the prediction of PFS, while with the CDL method, PSI achieved AUC scores of 0.995 (*P* = 0.000, 95% CI, 0.984–1.000) and 0.734 (*P* = 0.083, 95% CI, 0.479–0.989), respectively (AUC of the training set was calculated only based on data pertaining to the 64 patients). Table 2 shows accuracy and prognosis results. In the training set, the sensitivity of the WSDL method was superior to that of the CDL method (86.7% vs 73.3%, *P* = 0.048), while the methods showed the same specificity (100%). Due to the small number of patients in the test set, a comparison was not feasible.

**Table 2:** Deep learning feature-based detection efficiency and prognosis prediction

	Training set with WSDL (*n* = 64)	Test set with WSDL (*n* = 20)	Training set with CDL (*n* = 64)	Test set with CDL (*n* = 20)
Sensitivity	86.67%	87.50%	73.33%	62.5%
Specificity	100%	83.33%	100%	83.33%
Accuracy	93.75%	85.00%	87.50%	75.00%
2-year PFS (PSI > 1)	34.6% ± 9.3%	33.3% ± 15.7%	36.4% ± 10.3%	28.6% ± 17.1%
2-year PFS (PSI < 1)	92.1% ± 4.4%	90.9% ± 8.7%	85.7% ± 5.4%	84.6% ± 10.0%
5-year PFS (PSI > 1)	3.8% ± 3.8%	22.2% ± 13.9%	4.5% ± 4.4%	28.6% ± 17.1%
5-year PFS (PSI < 1)	92.1% ± 4.4%	90.9% ± 8.7%	77.1% ± 9.5%	74.0% ± 13.2%

Abbreviations: PFS, progression-free survival; PSI, prediction similarity index; WSDL, weakly supervised deep learning; CDL, conventional deep learning

**Fig. 5:**
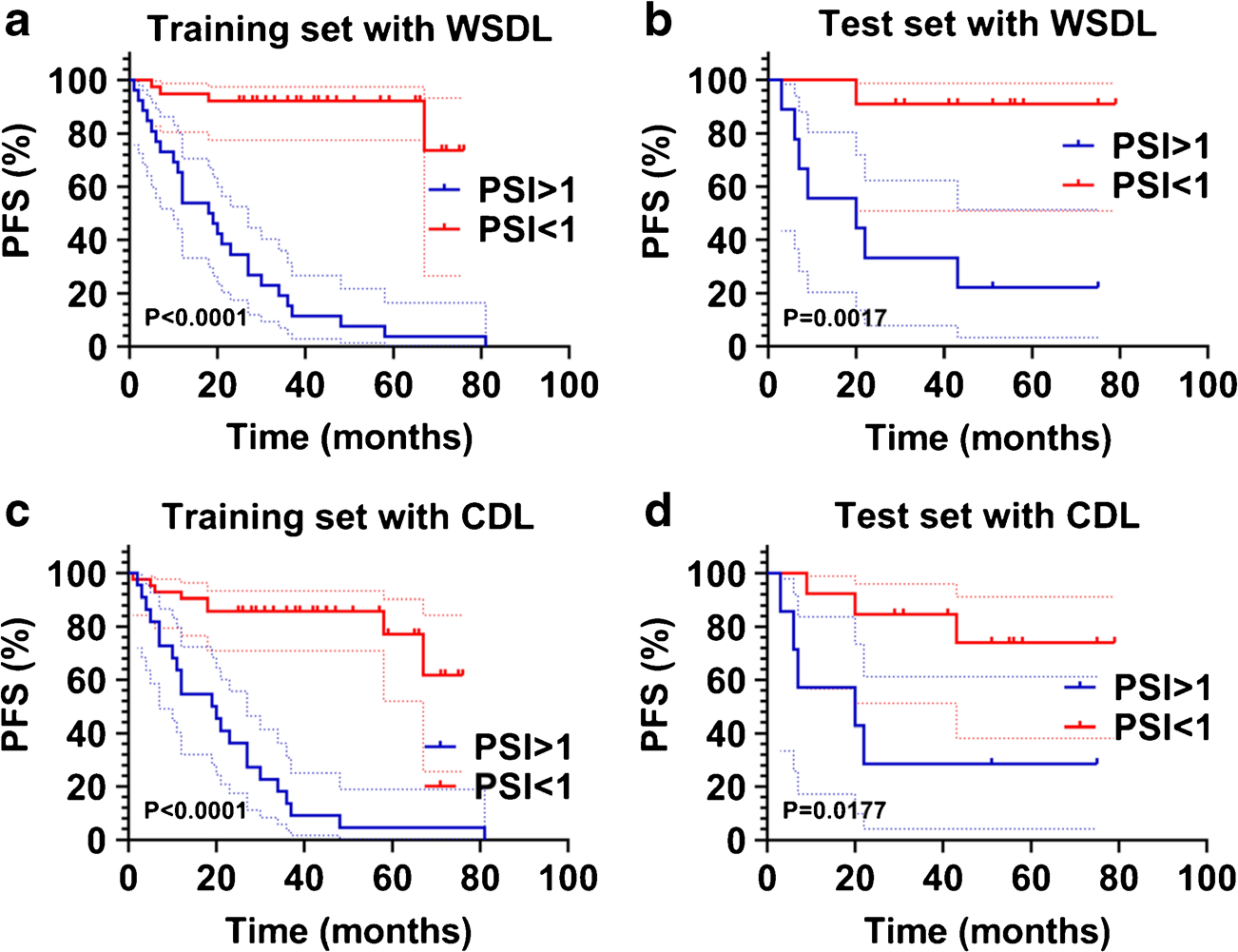
Kaplan–Meier estimates of PFS in the training (A) and test (B) sets of patients with high and low PSI. PFS, progression-free survival; PSI, prediction similarity index; WSDL, weakly supervised deep learning; CDL, conventional deep learning

According to PSI, patients were divided into two groups: PSI > 1 and PSI < 1. The Kaplan–Meier survival analysis method was used to compare differences in PFS between the groups. We observed that patients with low PSI (PSI < 1) showed good prognosis and long PFS, while those with high PSI (PSI > 1) showed poor prognosis and short PFS. Figure 5 shows the Kaplan–Meier curves of PFS according to PSI. The extracted PSI was able to segregate patients in the training set with different PFS in case of both the WSDL (*P* < 0.0001) and CDL (*P* < 0.0001) methods (Fig. 5A and C). Similarly, in the test set, the WSDL (*P* = 0.0017) and CDL (*P* = 0.0177) methods could distinguish patients with different PFS (Fig. 5B and D).

### Predictive value of other clinical and imaging parameters and integrated analysis

Major clinical factors, such as gender, serum lactate dehydrogenase levels, ECOG score, β2-microglobulin levels, and Epstein–Barr virus DNA, were significantly associated with PFS in univariate analysis. Conventional imaging parameters, including PET/CT-based Ann Arbor stage, MTV, and TLG, were also significantly associated with PFS in univariate analysis (refer to Table 3 for more details). Furthermore, we combined PSI with these clinical parameters to analyze the prognosis of ENKTL using the multivariate Cox proportional hazard model. We found that PSI was the only independent significant predictor of PFS. The WSDL method (HR, 15.183; 95% CI, 5.479–42.077; *P* = 0.000) achieved better PFS prognosis than the CDL method (HR, 7.857; 95% CI, 3.276–18.843; P = 0.000) after adjustment for various cofactors, as listed above.

**Table 3:** Univariate analysis involving patients with follow-up data

Characteristics	Training cohort (*n* = 64)		Test cohort (*n* = 20)		Total (*n* = 84)	
	Cutoff value	*P*	Cutoff value	*P*	Cutoff value	*P*
Gender	M/F	0.100	M/F	0.017	M/F	0.010
Age	60	0.184	60	0.041	60	0.742
Serum LDH	169* (0.092)	0.263	223* (0.137)	0.065	231.5 (0.019)	0.000
ECOG score	0/1/2/3/4	0.057	0/1/2/3/4	0.023	0/1/2/3/4	0.005
Ki67	60%* (0.767)	0.548	80%* (0.665)	0.870	70%* (0.970)	0.809
β2-microglobulin	188* (0.076)	0.387	454* (0.248)	0.328	820 (0.040)	0.001
EBV DNA	+/−	0.018	+/−	0.012	+/−	0.001
Ann Arbor stage	I–II/III–IV	0.000	I–II/III–IV	0.000	I–II/III–IV	0.000
B symptoms	+/−	0.300	+/−	0.441	+/−	0.193
PSI with CDL	1	0.000	1	0.018	1	0.000
PSI with WSDL	1	0.000	1	0.002	1	0.000
SUVmax	11.1* (0.382)	0.876	15.05* (0.418)	0.880	12.25* (0.218)	0.871
SUVmean	6.35* (0.453)	0.927	8.6* (0.298)	0.312	6.875* (0.249)	0.677
MTV	18.04 (0.002)	0.000	15.695* (0.165)	0.415	25.325 (0.001)	0.000
TLG	94.738 (0.004)	0.000	124.133* (0.316)	0.415	109.952 (0.006)	0.001

*Median value

Abbreviations: M, male; F, female; +, positive; −, negative; PFS, progression-free survival; PSI, prediction similarity index; WSDL, weakly supervised deep learning; CDL, conventional deep learning; LDH, lactate dehydrogenase; ECOG, Eastern Cooperative Oncology Group; EB virus, Epstein–Barr virus; SUV, standardized uptake value; MTV, metabolic tumor volume; TLG, total lesion glycolysis

## Discussion

The prognosis of high-risk ENKTL patients is generally poor [32, 38], and treating such patients is thus challenging. Although new regimes have been proposed, the response remains suboptimal due to strong disease heterogeneity [38]. Prognostic index of natural killer lymphoma (PINK) is a well-established index based on age, serum lactate dehydrogenase level, performance status, and disease stage. The PINK model [39] is based on clinical information; patients with the same PINK score could even show different prognosis. As a clinical molecular imaging method, ^18^F-FDG PET/CT shows good potential to help stratify patients and optimize prognosis for the treatment of many types of cancers [9, 40–42]. However, considering the low incidence of ENKTL, the potential of this method for predicting the prognosis of ENKTL remains poorly explored. Conventional ^18^F-FDG PET/CT-related parameters, such as SUVmax, SUVmean, MTV, and TGL, have been found to show a correlation with survival, but the results have been debatable [30, 31, 36, 43]. These parameters cannot facilitate a comprehensive image-based analysis of tumors and cannot be integrated in hematological guidelines [44] because prospective studies with larger cohort of patients and methodological harmonization are needed [45]. Our univariate analysis indicated that SUVmax and SUVmean were not related to prognosis, while MTV and TGL were related to prognosis. However, multivariate analyses indicated that none of them were associated with prognosis. Considering the rarity of ENKTL, it is difficult to predict its prognosis, particularly in small cohort of patients.

Considering the potential of AI in facilitating data analyses to discover useful information, we aimed to develop and validate AI methods to overcome the restriction of limited data availability and to explore the prognostic value of ^18^F-FDG PET/CT in ENKTL. We herein proposed an AI model that could utilize incomplete or missing follow-up data to enhance the prediction potential of deep learning methods. This improved prediction power of AI led to the extraction of feature maps from ^18^F-FDG PET/CT as effective surrogates for prognosis prediction in patients with ENKTL. Furthermore, the method could automatically discover characteristic features in metabolic imaging. Our results confirmed the benefits of AI for comprehensive imaging analyses, wherein the proposed PSI was better than conventional clinical parameters and other PET-related parameters for prognosis prediction.

AI methods tend to be biased toward texture rather than shape, while human cognitive processes function in the opposite manner [46]. Conventional ^18^F-FDG PET/CT-related parameters, such as Ann Arbor stage, SUVmax, SUVmean, MTV, and TGL, have been already covered within the AI framework, and they reportedly have inferior predictive performance than deep learning methods [47]. The current developments occurring within the field of AI can add value to conventional PET analyses. To avoid redundancy and correlation of tested data and to lower the number of parameters tested in view of the limited size of our cohort, Ann Arbor stage, MTV, and TLG were not included in multivariate analysis, although they were found to be related to prognosis in univariate analysis. For multivariate analysis, clinical prognostic factors and PSI were included. PSI eventually emerged to be the only independent predictor of PFS.

Despite their potential, the application of AI-based methods to clinical trials remains challenging due to limited sample sizes. Deep learning research is particularly difficult for rare diseases such as ENKTL. Moreover, not all recruited patients can be finally enrolled due to missing or incomplete follow-up. Therefore, we developed a WSDL method in an attempt to solve this problem. During the training of WSDL, implicit labels are generated by exploring similarities among patients, and this diversity can be captured by a deep neural network. Most supervised data augmentation methods have been developed by using unlabeled data for regularization under particular distributional assumptions, such as cluster or smoothness assumption [48]. However, the performance of such a model can be considerably deteriorated if the real data distribution violates the assumed distribution [14]. In this study, the proposed WSDL method with integrated PNU strategy did not make additional assumptions about data distribution; therefore, the performance of prognosis prediction was efficiently and robustly improved. We conducted a pilot study to reutilize the data without follow-up information to boost the prediction accuracy of patient survival; consequently, the advantages of the proposed WSDL method were confirmed in our test set. By employing WSDL, prognoses of patients in the test set could be significantly differentiated, and the results were better than on using CDL. Therefore, the proposed WSDL method may act as a practical tool for developing individualized treatment strategies using clinical trial data.

Tumor heterogeneity in baseline PET/CT images may allow better signature characterization and improve prediction of therapy response and survival in malignant tumors [49, 50]. Ko et al. [49] investigated whether the textural features of pretreatment ^18^F-FDG PET images could predict the prognosis for ENKTL; they reported that dissimilarity and low-intensity short-zone emphasis were significant predictors of disease progression in patients with ENKTL and were able to improve their prognostic stratification. However, there were only 17 patients in this retrospective study and details pertaining to the regimen were not mentioned. In our study, PSI was validated as a potential index for risk stratification and future management of patients with ENKTL. Compared with texture analyses, the results of deep learning are more difficult to interpret. Deep learning–based radiomics studies [9] evidently draw several image-based texture parameters and the significance of many of them cannot be explained in a clinical perspective; this hinders the application in clinical routine. In addition to the proposed PSI, we also visualized the extracted features as strips of feature maps. Although these maps did not give us an in-depth insight into physiological interpretation, they did give us an additional view of recommendations derived from the black box, and the different activation patterns may facilitate quality control in practice. The feature maps were composed of multiple features, and, therefore, they contained more information than a single scalar value of PSI. An increase in the dimension of the features may improve prediction but may lead to overfitting. On the other hand, a single scalar value is convenient for clinical interpretation. Therefore, it may be practical to consider both PSI values and feature maps to gather better, more robust information.

This study had several limitations. First, although we employed WSDL to enhance data utilization, the sample size was still small, which may reduce the test power and predictive ability of deep learning methods. Similar to other studies based on rare diseases, the difference between overall survival and PFS was not great, and we did not perform overall survival-related survival analysis. We only performed survival analysis based on PFS. Second, tumors were outlined by a specialist in medical radiology and nuclear medicine. As with previous studies, interobserver variations may exist in the manual delineation and may influence the reported results [9]. Nevertheless, deep learning methods can automatically learn features included in the hidden layers of neural networks from imaging data, and they are less sensitive to segmentation variations [51, 52]. Third, study data were collected from a single center, and external validation is thus necessary to validate our findings. Finally, potential patient selection biases may exist because of the retrospective nature of this study.

To summarize, our proposed WDSL method was able to utilize incomplete or missing follow-up data to improve survival prediction. Deep learning involving ^18^F-FDG PET/CT provides an effective approach for prognosis prediction in patients with ENKTL. The identified feature maps and PSI may potentially assist the stratification of patients in therapy. Future prospective studies with external validation are nevertheless warranted to validate our findings.

## Supplementary information

Fig. S1 Visualization of feature maps (16 × 8) representing 128 features extracted by the proposed WSDL method in the relapse group of the training set. PSI results with incorrect predictions have been marked by red boxes. (PNG 56791 kb)

High resolution image (TIFF 4044 kb)

Fig. S2 Visualization of feature maps (16 × 8) representing 128 features extracted by the proposed WSDL method in the non-relapse group of the training set. (PNG 76302 kb)

High resolution image (TIFF 4685 kb)

Fig. S3 Visualization of feature maps (16 × 8) representing 128 features extracted by the proposed WSDL method in the patients with incomplete or missing follow-up data. (PNG 9483 kb)

High resolution image (TIFF 1946 kb)

Fig. S4 Visualization of feature maps (16 × 8) representing 128 features extracted by the proposed CDL method in the test set. PSI results with incorrect predictions have been marked by red boxes. (PNG 53166 kb)

High resolution image (TIFF 3189 kb)

Fig. S5 Visualization of feature maps (16 × 8) representing 128 features extracted by the proposed CDL method in the relapse group of the training set. PSI results with incorrect predictions have been marked by red boxes. (PNG 63781 kb)

High resolution image (TIFF 4445 kb)

Fig. S6 Visualization of feature maps (16 × 8) representing 128 features extracted by the proposed CDL method in the non-relapse group of the training set. (PNG 84520 kb)

High resolution image (TIFF 5139 kb)

ESM 1 (DOCX 2655 kb)

## References

[1] Hatt M, Le Rest CC, Tixier F, Badic B, Schick U, Visvikis D (2019). Radiomics: data are also images. J Nucl Med.

[2] Visvikis D, Cheze Le Rest C, Jaouen V, Hatt M (2019). Artificial intelligence, machine (deep) learning and radio(geno)mics: definitions and nuclear medicine imaging applications. Eur J Nucl Med Mol Imaging.

[3] Li L, Qin L, Xu Z, Yin Y, Wang X, Kong B (2020). Using artificial intelligence to detect COVID-19 and community-acquired pneumonia based on pulmonary CT: evaluation of the diagnostic accuracy. Radiology..

[4] Esteva A, Kuprel B, Novoa RA, Ko J, Swetter SM, Blau HM (2017). Dermatologist-level classification of skin cancer with deep neural networks. Nature..

[5] Kermany DS, Goldbaum M, Cai W, Valentim CCS, Liang H, Baxter SL (2018). Identifying medical diagnoses and treatable diseases by image-based deep learning. Cell..

[6] Blanc-Durand P, Campedel L, Mule S, Jegou S, Luciani A, Pigneur F (2020). Prognostic value of anthropometric measures extracted from whole-body CT using deep learning in patients with non-small-cell lung cancer. Eur Radiol.

[7] Xu Y, Hosny A, Zeleznik R, Parmar C, Coroller T, Franco I (2019). Deep learning predicts lung cancer treatment response from serial medical imaging. Clin Cancer Res.

[8] Tang Z, Xu Y, Jin L, Aibaidula A, Lu J, Jiao Z (2020). Deep learning of imaging phenotype and genotype for predicting overall survival time of glioblastoma patients. IEEE Trans Med Imaging.

[9] Peng H, Dong D, Fang MJ, Li L, Tang LL, Chen L (2019). Prognostic value of deep learning PET/CT-based radiomics: potential role for future individual induction chemotherapy in advanced nasopharyngeal carcinoma. Clin Cancer Res.

[10] Litjens G, Kooi T, Bejnordi BE, Setio AAA, Ciompi F, Ghafoorian M (2017). A survey on deep learning in medical image analysis. Med Image Anal.

[11] Razzak MI, Naz S, Zaib A. Deep learning for medical image processing: overview, challenges and the future. Classification in BioApps. 2018:323–50.

[12] Karimi D, Nir G, Fazli L, Black PC, Goldenberg L, Salcudean SE (2020). Deep learning-based Gleason grading of prostate cancer from histopathology images-role of multiscale decision aggregation and data augmentation. IEEE J Biomed Health Inform.

[13] Leunens G, Verstraete J, Van den Bogaert W, Van Dam J, Dutreix A, van der Schueren E (1992). Human errors in data transfer during the preparation and delivery of radiation treatment affecting the final result: “garbage in, garbage out”. Radiother Oncol.

[14] Krijthe JH, Loog M (2017). Robust semi-supervised least squares classification by implicit constraints. Pattern Recogn.

[15] Sakai T, MCdP, Niu G, Sugiyama M. Semi-supervised classification based on classification from positive and unlabeled data. The 34th International Conference on Machine Learning. Sydney, Australia; 2017;2998–3006.

[16] Yu-Feng Li Z-HZ (2015). Towards making unlabeled data never hurt. IEEE Trans Pattern Anal Mach Intell.

[17] Lee J, Suh C, Park YH, Ko YH, Bang SM, Lee JH (2006). Extranodal natural killer T-cell lymphoma, nasal-type: a prognostic model from a retrospective multicenter study. J Clin Oncol.

[18] Au WY, Ma SY, Chim CS, Choy C, Loong F, Lie AK (2005). Clinicopathologic features and treatment outcome of mature T-cell and natural killer-cell lymphomas diagnosed according to the World Health Organization classification scheme: a single center experience of 10 years. Ann Oncol.

[19] Li CC, Tien HF, Tang JL, Yao M, Chen YC, Su IJ (2004). Treatment outcome and pattern of failure in 77 patients with sinonasal natural killer/T-cell or T-cell lymphoma. Cancer..

[20] The world health organization classification of malignant lymphomas in japan: incidence of recently recognized entities. Lymphoma Study Group of Japanese Pathologists. Pathol Int. 2000;50:696–702. 10.1046/j.1440-1827.2000.01108.x 11012982

[21] Chen CY, Yao M, Tang JL, Tsay W, Wang CC, Chou WC (2004). Chromosomal abnormalities of 200 Chinese patients with non-Hodgkin’s lymphoma in Taiwan: with special reference to T-cell lymphoma. Ann Oncol.

[22] Chan WK, Au WY, Wong CY, Liang R, Leung AY, Kwong YL (2010). Metabolic activity measured by F-18 FDG PET in natural killer-cell lymphoma compared to aggressive B- and T-cell lymphomas. Clin Nucl Med.

[23] Khong PL, Pang CB, Liang R, Kwong YL, Au WY (2008). Fluorine-18 fluorodeoxyglucose positron emission tomography in mature T-cell and natural killer cell malignancies. Ann Hematol.

[24] Moon SH, Cho SK, Kim WS, Kim SJ, Chan Ahn Y, Choe YS (2013). The role of 18F-FDG PET/CT for initial staging of nasal type natural killer/T-cell lymphoma: a comparison with conventional staging methods. J Nucl Med.

[25] Zhou X, Lu K, Geng L, Li X, Jiang Y, Wang X (2014). Utility of PET/CT in the diagnosis and staging of extranodal natural killer/T-cell lymphoma: a systematic review and meta-analysis. Medicine (Baltimore).

[26] Casulo C, Schoder H, Feeney J, Lim R, Maragulia J, Zelenetz AD (2013). 18F-fluorodeoxyglucose positron emission tomography in the staging and prognosis of T cell lymphoma. Leuk Lymphoma.

[27] Fujiwara H, Maeda Y, Nawa Y, Yamakura M, Ennishi D, Miyazaki Y (2011). The utility of positron emission tomography/computed tomography in the staging of extranodal natural killer/T-cell lymphoma. Eur J Haematol.

[28] Wu HB, Wang QS, Wang MF, Li HS, Zhou WL, Ye XH (2010). Utility of 18F-FDG PET/CT for staging NK/T-cell lymphomas. Nucl Med Commun.

[29] Karantanis D, Subramaniam RM, Peller PJ, Lowe VJ, Durski JM, Collins DA (2008). The value of [(18)F]fluorodeoxyglucose positron emission tomography/computed tomography in extranodal natural killer/T-cell lymphoma. Clin Lymphoma Myeloma.

[30] Suh C, Kang YK, Roh JL, Kim MR, Kim JS, Huh J (2008). Prognostic value of tumor 18F-FDG uptake in patients with untreated extranodal natural killer/T-cell lymphomas of the head and neck. J Nucl Med.

[31] Khong PL, Huang B, Lee EY, Chan WK, Kwong YL (2014). Midtreatment 18F-FDG PET/CT scan for early response assessment of SMILE therapy in natural killer/T-cell lymphoma: a prospective study from a single center. J Nucl Med.

[32] Guo R, Xu P, Xu H, Miao Y, Li B (2020). The predictive value of pre-treatment 18F-FDG PET/CT on treatment outcome in early-stage extranodal natural killer/T-cell lymphoma. Leuk Lymphoma.

[33] Bai B, Huang HQ, Cai QC, Fan W, Wang XX, Zhang X (2013). Predictive value of pretreatment positron emission tomography/computed tomography in patients with newly diagnosed extranodal natural killer/T-cell lymphoma. Med Oncol.

[34] Chang Y, Fu X, Sun Z, Xie X, Wang R, Li Z (2017). Utility of baseline, interim and end-of-treatment (18)F-FDG PET/CT in extranodal natural killer/T-cell lymphoma patients treated with L-asparaginase/pegaspargase. Sci Rep.

[35] Jiang C, Zhang X, Jiang M, Zou L, Su M, Kosik RO (2015). Assessment of the prognostic capacity of pretreatment, interim, and post-therapy (18)F-FDG PET/CT in extranodal natural killer/T-cell lymphoma, nasal type. Ann Nucl Med.

[36] Jiang C, Su M, Kosik RO, Zou L, Jiang M, Tian R (2015). The Deauville 5-point scale improves the prognostic value of interim FDG PET/CT in extranodal natural killer/T-cell lymphoma. Clin Nucl Med.

[37] He K, Zhang X, Ren S, Sun J. Deep residual learning for image recognition. 2016 IEEE Conference on Computer Vision and Pattern Recognition (CVPR); 2016;770–8.

[38] Tse E, Kwong YL (2017). The diagnosis and management of NK/T-cell lymphomas. J Hematol Oncol.

[39] Kim SJ, Yoon DH, Jaccard A, Chng WJ, Lim ST, Hong H (2016). A prognostic index for natural killer cell lymphoma after non-anthracycline-based treatment: a multicentre, retrospective analysis. Lancet Oncol.

[40] Cheng NM, Hsieh CE, Fang YD, Liao CT, Ng SH, Wang HM (2020). Development and validation of a prognostic model incorporating [(18)F]FDG PET/CT radiomics for patients with minor salivary gland carcinoma. EJNMMI Res.

[41] Senjo H, Hirata K, Izumiyama K, Minauchi K, Tsukamoto E, Itoh K (2020). High metabolic heterogeneity on baseline 18FDG-PET/CT scan as a poor prognostic factor for newly diagnosed diffuse large B-cell lymphoma. Blood Adv.

[42] Pinho DF, King B, Xi Y, Albuquerque K, Lea J, Subramaniam RM (2020). Value of Intratumoral metabolic heterogeneity and quantitative (18)F-FDG PET/CT parameters in predicting prognosis for patients with cervical cancer. AJR Am J Roentgenol.

[43] Kim CY, Hong CM, Kim DH, Son SH, Jeong SY, Lee SW (2013). Prognostic value of whole-body metabolic tumour volume and total lesion glycolysis measured on (18)F-FDG PET/CT in patients with extranodal NK/T-cell lymphoma. Eur J Nucl Med Mol Imaging.

[44] Barrington SF, Mikhaeel NG, Kostakoglu L, Meignan M, Hutchings M, Mueller SP (2014). Role of imaging in the staging and response assessment of lymphoma: consensus of the international conference on malignant lymphomas imaging working group. J Clin Oncol.

[45] Aide N, Lasnon C, Veit-Haibach P, Sera T, Sattler B, Boellaard R (2017). EANM/EARL harmonization strategies in PET quantification: from daily practice to multicentre oncological studies. Eur J Nucl Med Mol Imaging.

[46] Kubilius J, Bracci S, Op de Beeck HP (2016). Deep neural networks as a computational model for human shape sensitivity. PLoS Comput Biol.

[47] Baek S, He Y, Allen BG, Buatti JM, Smith BJ, Tong L (2019). Deep segmentation networks predict survival of non-small cell lung cancer. Sci Rep.

[48] Chapelle O, Zien A. Semi-supervised classification by low density separation. AISTATS. 2005:57–64.

[49] Ko KY, Liu CJ, Ko CL, Yen RF (2016). Intratumoral heterogeneity of pretreatment 18F-FDG PET images predict disease progression in patients with nasal type extranodal natural killer/T-cell lymphoma. Clin Nucl Med.

[50] Gao J, Huang X, Meng H, Zhang M, Zhang X, Lin X (2020). Performance of multiparametric functional imaging and texture analysis in predicting synchronous metastatic disease in pancreatic ductal adenocarcinoma patients by hybrid PET/MR: initial experience. Front Oncol.

[51] LeCun Y, Bengio Y, Hinton G (2015). Deep learning. Nature..

[52] Tajbakhsh N, Shin JY, Gurudu SR, Hurst RT, Kendall CB, Gotway MB (2016). Convolutional neural networks for medical image analysis: full training or fine tuning?. IEEE Trans Med Imaging.

