# Supplementary figures and images for: Weakly supervised deep learning for determining the prognostic value of 18F-FDG PET/CT in extranodal natural killer/T cell lymphoma, nasal type
Source: Eur J Nucl Med Mol Imaging. 2021 Feb 20;48(10):3151–61. doi: 10.1007/s00259-021-05232-3 (PMC7896833; doi:10.1007/s00259-021-05232-3)

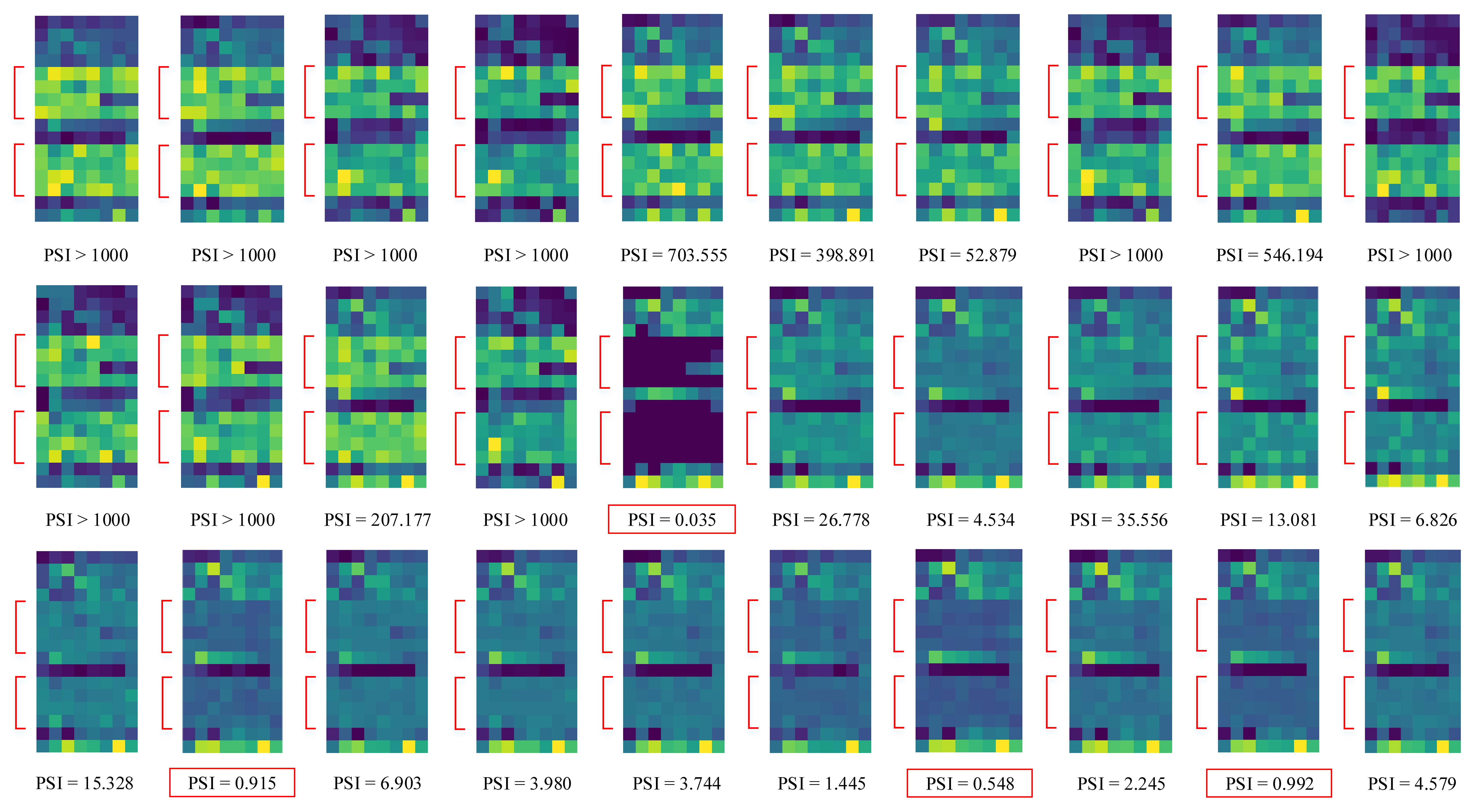

Supplement: Supplementary file 1 — Visualization of feature maps (16 × 8) representing 128 features extracted by the proposed WSDL method in the relapse group of the training set. PSI results with incorrect predictions have been marked by red boxes. (PNG 56791 kb) [file 259_2021_5232_Fig6_ESM.png]

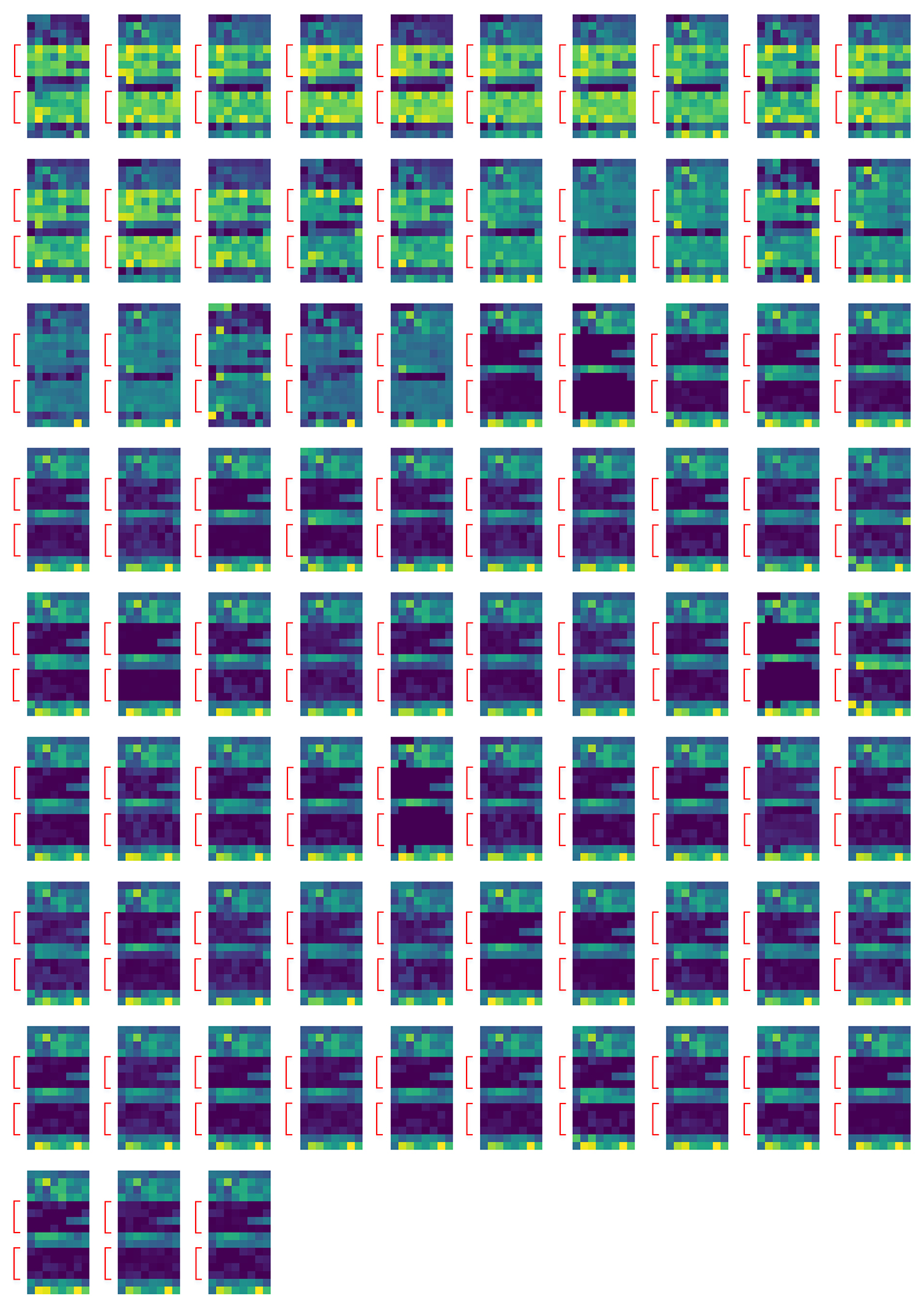

Supplement: Supplementary file 5 — Visualization of feature maps (16 × 8) representing 128 features extracted by the proposed WSDL method in the patients with incomplete or missing follow-up data. (PNG 9483 kb) [file 259_2021_5232_Fig8_ESM.png]

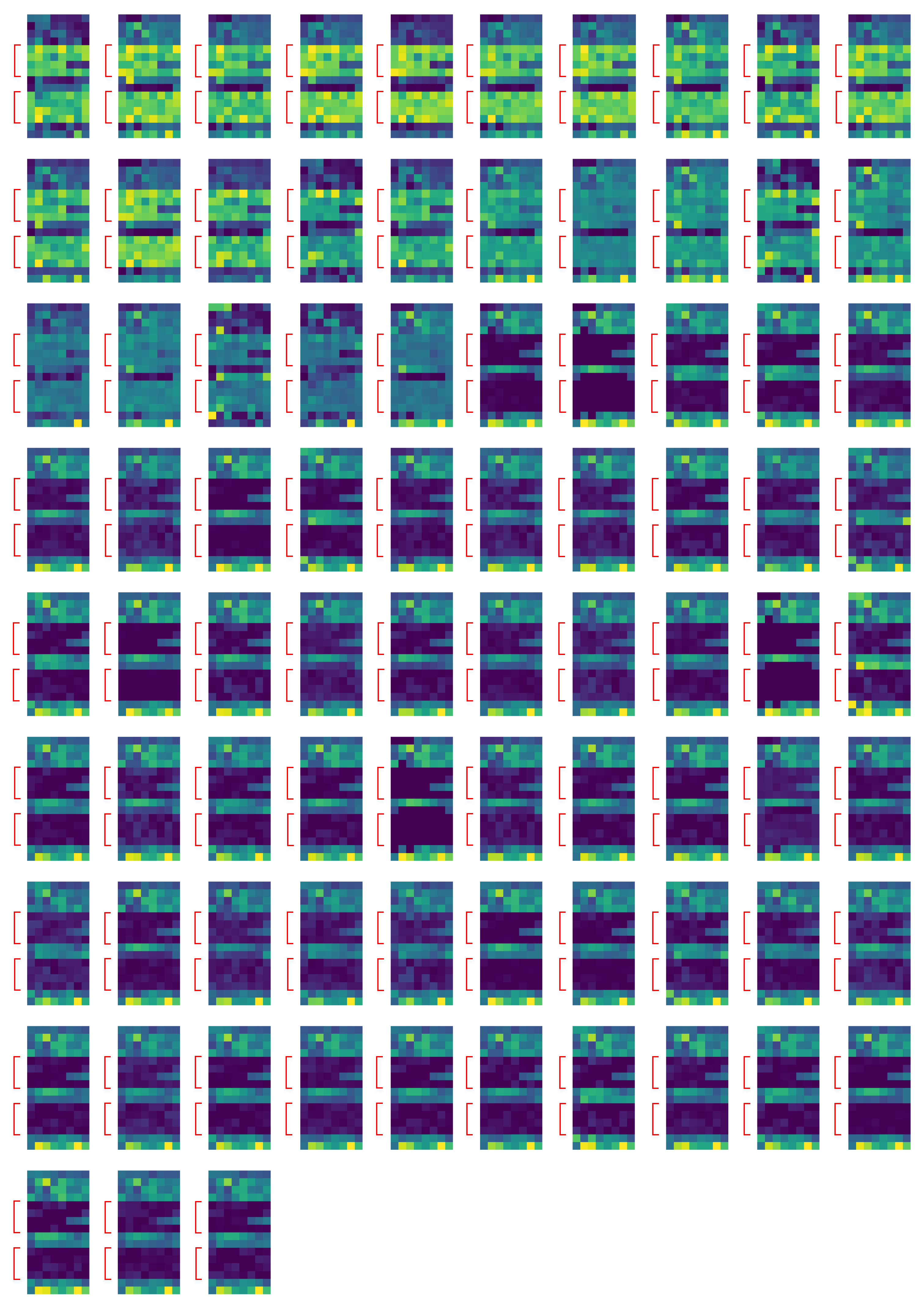

Supplement: Supplementary file 6 — High resolution image (TIFF 1946 kb) [file 259_2021_5232_MOESM3_ESM.tiff]

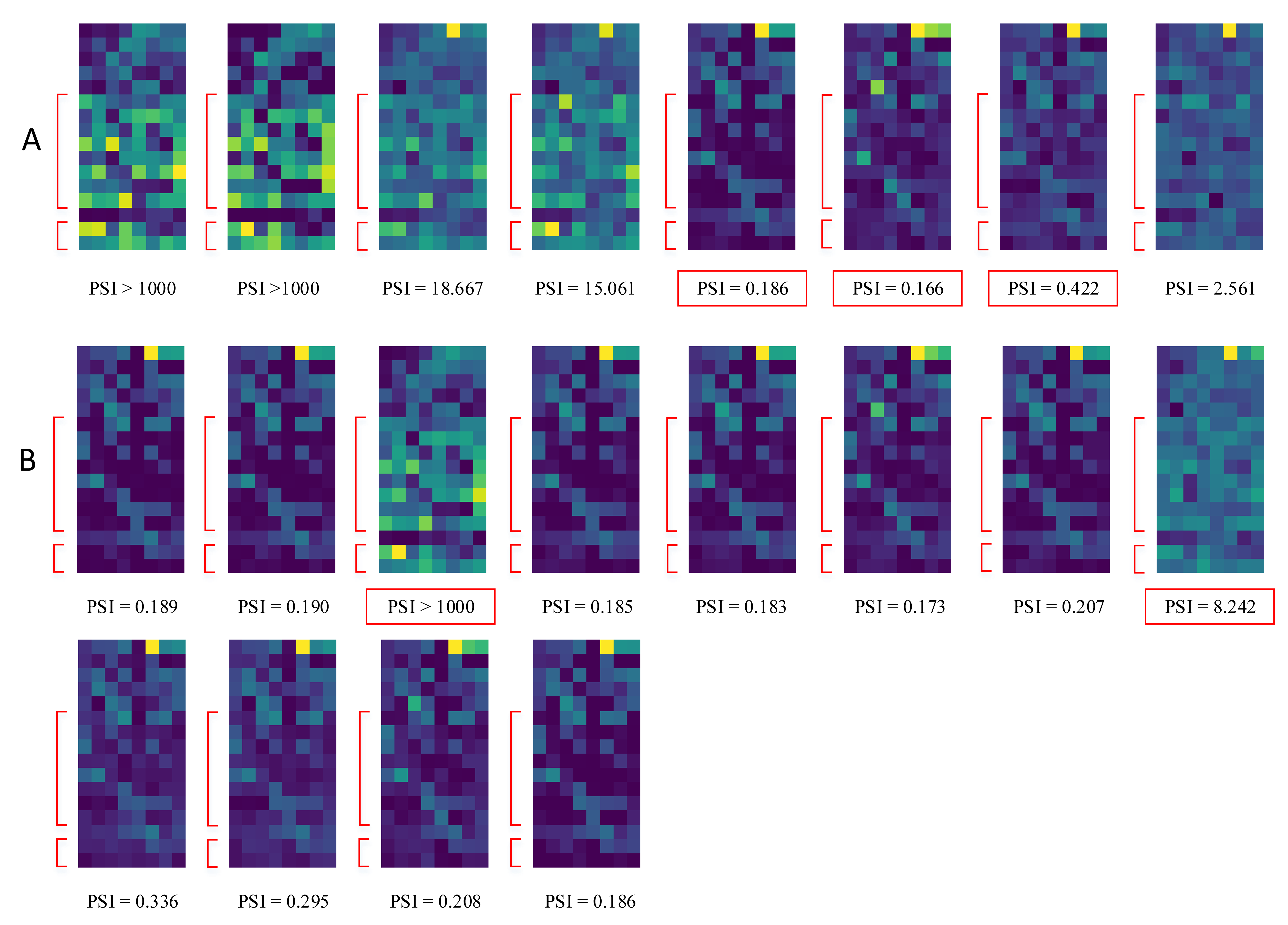

Supplement: Supplementary file 7 — Visualization of feature maps (16 × 8) representing 128 features extracted by the proposed CDL method in the test set. PSI results with incorrect predictions have been marked by red boxes. (PNG 53166 kb) [file 259_2021_5232_Fig9_ESM.png]

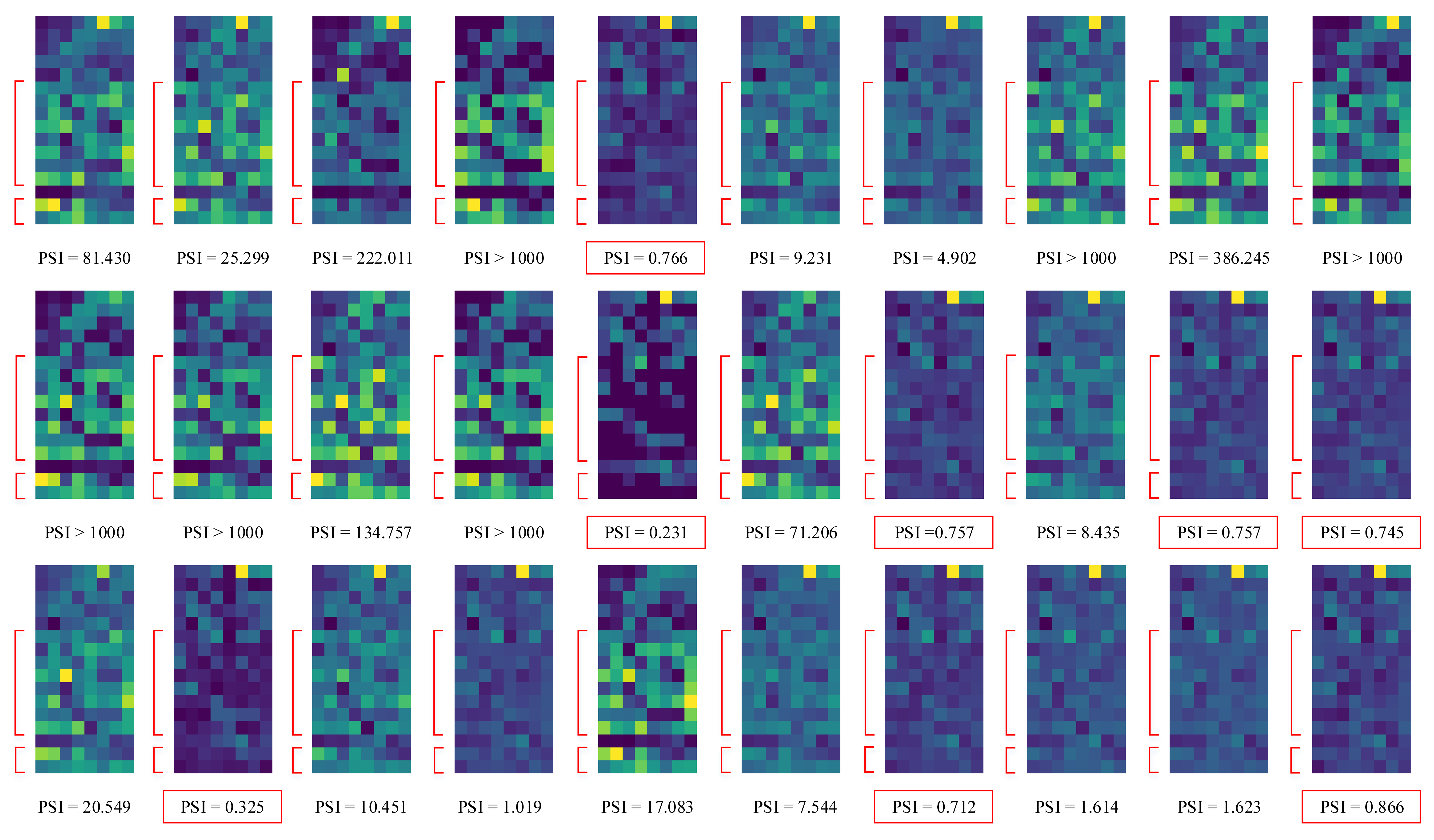

Supplement: Supplementary file 9 — Visualization of feature maps (16 × 8) representing 128 features extracted by the proposed CDL method in the relapse group of the training set. PSI results with incorrect predictions have been marked by red boxes. (PNG 63781 kb) [file 259_2021_5232_Fig10_ESM.png]
