# Supplementary material for: Weakly supervised deep learning for determining the prognostic value of 18F-FDG PET/CT in extranodal natural killer/T cell lymphoma, nasal type
Source: Eur J Nucl Med Mol Imaging. 2021 Feb 20;48(10):3151–61. doi: 10.1007/s00259-021-05232-3 (PMC7896833; doi:10.1007/s00259-021-05232-3)
Supplement: Supplementary file 13 — (DOCX 2655 kb) [file 259_2021_5232_MOESM7_ESM.docx]

**Supplementary Materials**

**WSDL for feature extraction**

In this study, the WSDL method used 3D volumetric PET/CT images of segmented lesions as input, and the method included the following steps:


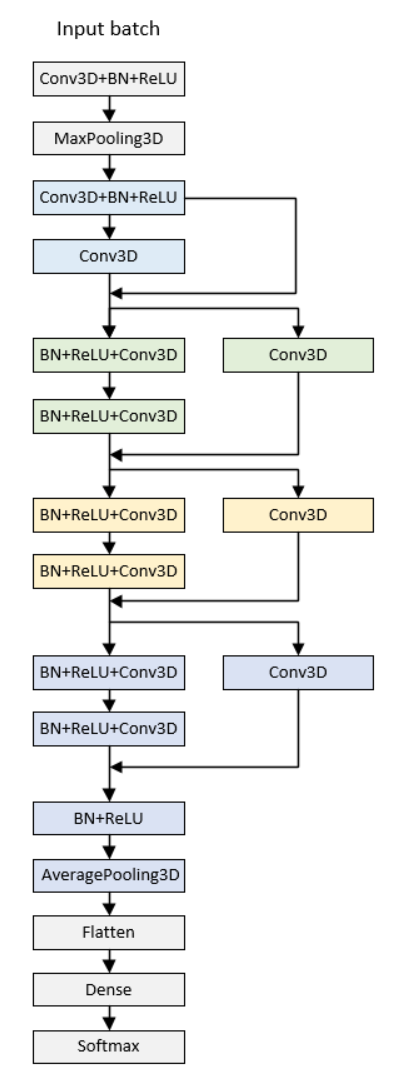
**Step 1:** In the first step, we used only labeled data to pretrain DCNNs. These pretrained DCNNs generated the preliminary feature representations of PET/CT images, giving insights into prognostic knowledge. DCNNs are a simplified version of the ResNet [1]; the detailed structure is shown in Figure I. Due to the Conv3D structure, DCNNs can extract features from 3D segmentation lesions of whole body volume rather than 2D slice information that ignores spatial correlation. Consequently, our 3D models showed a better, more robust performance in comparison with 2D-based methods.

Figure I. The proposed 10-layer 3D CNNs comprised several modules of Conv3D, Maxpooling3D, and BN layers. Conv3D, 3D convolution; BN, batch normalization; ReLU, Rectified Linear Units.

Inspired from the ResNet, we designed 10-layer 3D CNNs that comprised four residual convolutional layer groups consisting of 3D filters, all with a kernel size of 3 × 3 × 3. The number of kernels in each convolutional layer group was set to 16, 32, 64, and 128. Maxpooling3D (2 × 2) was used in the first layer, and the first convolutional layer group was composed of a classical Conv–ReLU–BN module. This was followed by three convolutional layer groups with the full preactivation BN–ReLU–Conv structure, which made training easier and improved generalization. We used Conv3D in the short-cut connection to down-sample the features and skip the regular max-pooling part. Due to overfitting and memory limitation, 3D Global Average Pooling was used in the final convolutional layer group. The final output features were flattened and passed to a dense-connected part with 128 neurons. The output was then carried to a dense layer of two neurons with Softmax activation for prognostic prediction.


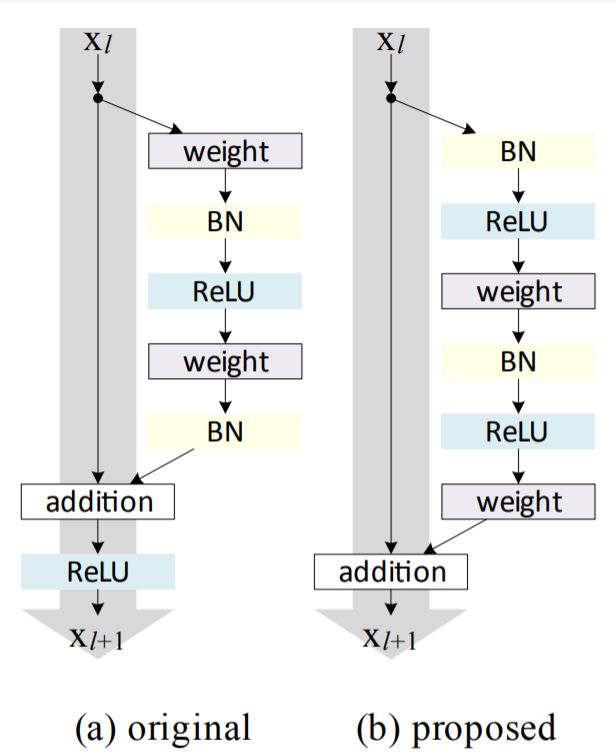


Figure II. The full preactivation Residual Unit compared with the original Residual Unit.

**Step 2:** The unlabeled data were then integrated and pretrained DCNNs were used to extract features from both labeled and unlabeled data. As shown in Figure III (B), 128 features were extracted from the output of the average pooling layer and normalized to [0,1]. The extracted features were used to construct the PNU classifier to generate implicit labels for unlabeled data. In this manner, more information was obtained to distinguish between the classes.


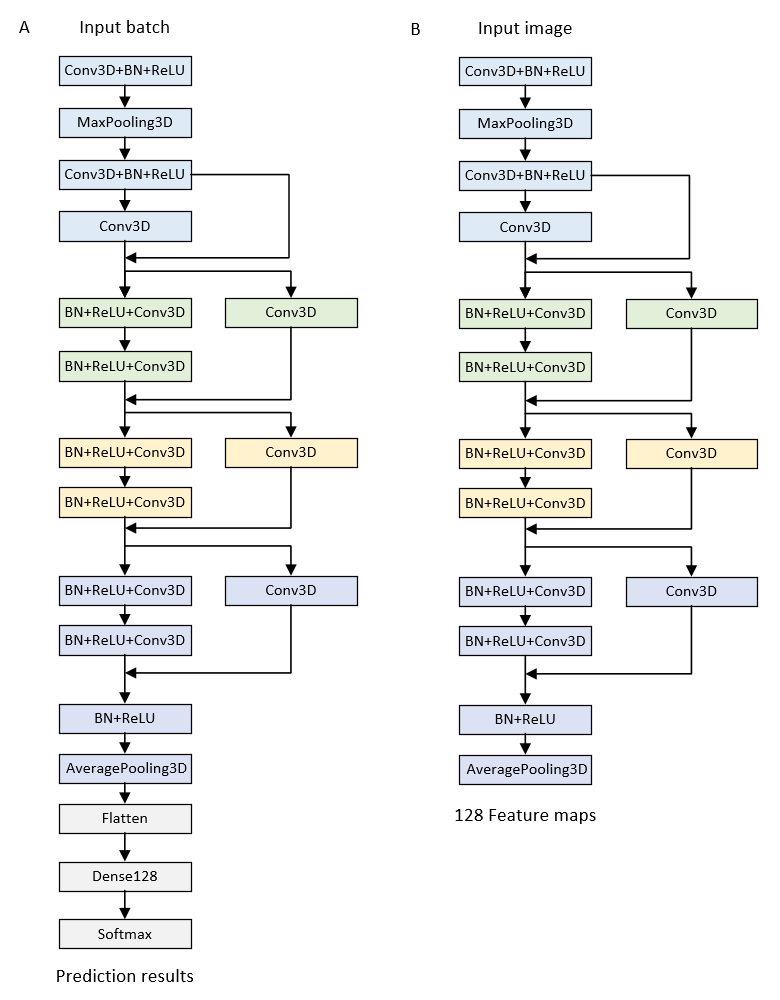
Figure III DCNN architectures. (A) DCNNs for images in the training step and (B) for images in the feature extraction step. Conv3D, 3D convolution; BN, batch normalization; ReLU, Rectified Linear Units.

**Step 3:** We used the PNU classification [3] method for enlarging our training data.


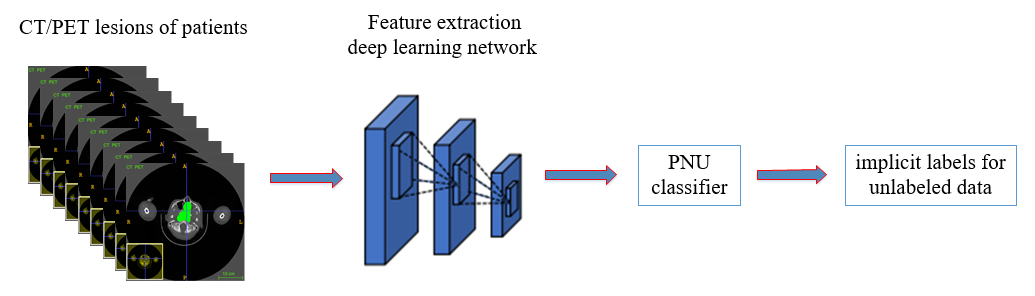
Figure IV The PNU classifier appeared midway in our WSDL method.

As shown in Figure IV, the PNU classifier was built to generate implicit labels for unlabeled data, and then these along with labeled data were input into DCNNs for retraining. The results of the PNU classification method are a critical step in the WSDL method and are not directly applied to predict therapeutic results.

**Step 4:** DCNNs were retrained using labeled and implicitly labeled data together to obtain the final model for prognostic prediction. The WSDL method enabled the model to maximize the utility of incomplete and missing follow-up data to improve survival prediction of ENKTL.

The summarized algorithm for the WSDL framework is as follows:

**Input: 3D volumetric image I of size width × height × depth.**

**Ensure: Image I is a rank 3 tensor**

**1: Train DCNNs with labeled data to obtain the baseline model**

**2: Use baseline DCNNs to extract features from labeled and unlabeled data**

**3: Build the PNU classifier to generate implicit labels for unlabeled data**

**4: Re-train DCNNs with labeled and unlabeled data to obtain the final model**

**Training settings**

Implementation details: DCNNs were constructed based on the network structure shown in Figure I. They were implemented using the Python Keras package with TensorFlow as the backend, and trained on a machine with Nvidia TITAN Xp with 12 GB memory. For the baseline model (CDL), the networks accepted input data of size 287 × 128 × 128 with a batch size of 2, while the batch size was set to 3 for retraining the final model (WSDL). We used Adam optimizer with a default learning rate of 0.001. Each model was trained for 30 epochs, and we stopped training early to avoid overfitting. We used class weight to balance the dataset, and the loss function was the weighted binary cross-entropy loss. Loss function formula:

$$J_{BCE}=\text{-}\sum[W_{0}\times\text{y}\times\log(\text{p)}+ W_{1}\times(1-y)\times\log(1-p)]/(N_{0}+N_{1})$$

$$W_{1}=N_{1}/2\times\text{（}N_{0}+N_{1})$$

$$W_{0}=\text{N}_{0}/2\times\text{（}N_{0}+N_{1})$$

where$N_{0}$and$N_{1}$are the data number of class0 and class1, respectively, while $W_{0}$and$W_{1}$act as the weight to balance the binary cross-entropy loss $J_{BCE}$.

For the PNU classifier, we used the Gaussian kernel model for the WSDL method. The class prior of labeled data was estimated based on the energy distance method [9]. The n-fold was set to 5 and the other parameter for the PNU method was set to the default value. Implementation details pertaining to the PNU method and Python codes are available here: https://github.com/t-sakai-kure/pywsl.

**Definition of Training Data**

Postoperative prediction can be regarded as a classification problem. In our experiments, patients with a positive response were given label 1, while those with a negative response were given label 0. In general, one-hot encoding is the standard approach to encode category labels. One-hot encoding is a process by which categorical variables are converted into a form that can be provided to ML algorithms to improve prediction performance. Figure V shows an example:


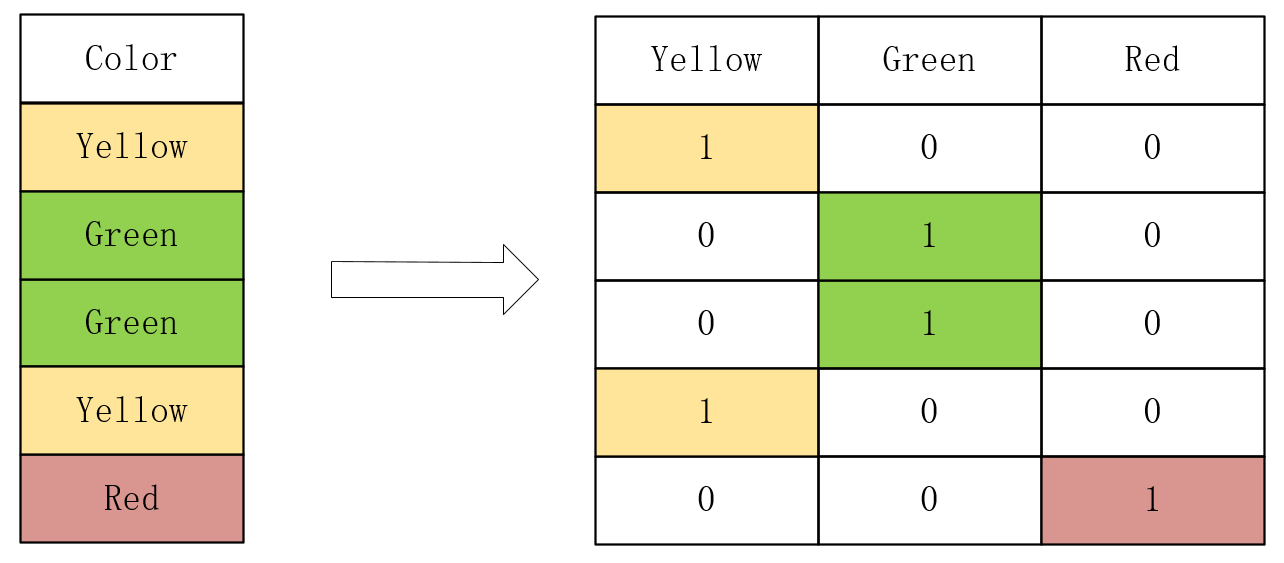
Figure V One-hot encoding creates new columns indicating the presence (or absence) of each possible value in the original data.

In the original dataset, “color” is a categorical variable with three labels: “Yellow”, “Green,’’ and “Red”. The corresponding one-hot encoding contains one column for each possible value, and one row for each row in the original dataset. Wherever the original value was “Yellow,” we insert 1 in the “Yellow” column, and if the original value was “Green,” we insert 1 in the “Green” column, and so on. One-hot encoding does not assume an ordering of the categories and works particularly well if there is no clear ordering in categorical data (“Green” is neither more nor less than “Yellow”). In our experiments, we used the label [0,1] for positive response patients and [1,0] for negative response patients.

**References**

1. He K, Zhang X, Ren S, Sun J. Deep Residual Learning for Image Recognition. 2016 IEEE Conference on Computer Vision and Pattern Recognition (CVPR); 2016. p. 770-8.

2. He K, Zhang X, Ren S, Sun J. Identity Mappings in Deep Residual Networks. ArXiv. 2016;abs/1603.05027.

3. Tomoya Sakai MCdP, Gang Niu, Masashi Sugiyama. Semi-Supervised Classification Based on Classification from Positive and Unlabeled Data. the 34 th International Conference on Machine Learning. Sydney, Australia; 2017. p. 2998-3006.

4. Kawakubo H, Plessis M, Sugiyama M. Computationally Efficient Class-Prior Estimation under Class Balance Change Using Energy Distance. IEICE Transactions on Information and Systems. 2016;E99.D:176-86.
